# Thyroid Hormone Changes in the Northern Area of Tianjin during the COVID-19 Pandemic

**DOI:** 10.1155/2022/5720875

**Published:** 2022-01-07

**Authors:** Dong Weiwei, Wu Bei, Wang Hong, Wu Cailan, Shao Hailin, Xu Donghong, Wang Xiaolai, Hao Zhaohu, Li Shijun, Tan Jian, Jia Qiang

**Affiliations:** ^1^Department of Nuclear Medicine, Tianjin Fourth Central Hospital, The Fourth Central Clinical School, Tianjin Medical University, Tianjin 300140, China; ^2^Department of Nuclear Medicine, Tianjin Medical University General Hospital, Tianjin Medical University, Tianjin 300052, China; ^3^Department of Nuclear Medicine, Tianjin Hospital, Tianjin 300211, China; ^4^Rehabilitation Medical Department, Tianjin Union Medical Center, Tianjin 300121, China; ^5^Department of Endocrinology Medicine, Tianjin Fourth Central Hospital, The Fourth Central Clinical School, Tianjin Medical University, Tianjin 300140, China; ^6^Department of Hematology, Tianjin Fourth Central Hospital, The Fourth Central Clinical School, Tianjin Medical University, Tianjin 300140, China

## Abstract

**Purpose:**

This study aimed to determine whether and how stress-induced thyroid hormone changes occur during the COVID-19 pandemic in the northern area of Tianjin.

**Methods:**

This study comprised two groups of study subjects in Tianjin: before (2019) and during (2020) the COVID-19 outbreak. Subjects were included if they had FT3, FT4, and TSH concentrations and thyroid TPOAb or TgAb information available. People who were pregnant, were lactating, or had mental illness were excluded. We used propensity score matching to form a cohort in which patients had similar baseline characteristics, and their anxiety level was measured by the Hamilton Anxiety Rating Scale (HAMA).

**Results:**

Among the 1395 eligible people, 224 in Group A and 224 in Group B had similar propensity scores and were included in the analyses. The detection rate of abnormal thyroid function was decreased in pandemic Group B (69.2% vs. 93.3%, *χ*^2^ = 42.725, *p* < 0.01), especially for hypothyroidism (14.29% vs. 35.71%, *χ*^2^ = 27.429, *p* < 0.01) and isolated thyroid-related antibodies (25.89% vs. 38.39%, *χ*^2^ = 8.023, *p* < 0.01). The level of FT4 (*z* = −2.821, *p* < 0.01) and HAMA score (7.63 ± 2.07 vs. 5.40 ± 1.65, *t* = 16.873, *p* < 0.01) went up in Group B; however, TSH (*z* = −5.238, *p* < 0.01), FT3 (*z* = −3.089, *p*=0.002), TgAb (*z* = −11.814, *p* < 0.01), and TPOAb (*z* = −9.299, *p* < 0.01) were lower, and HAMA was positive with FT3 (*r* = 0.208, *p* < 0.01) and FT4 (*r* = 0.247, *p* < 0.01).

**Conclusion:**

People in the northern area of Tianjin during the COVID-19 outbreak were at an increased risk of higher FT4, lower FT3, and lower TSH. The HAMA scores increased in emergency situations and were positively correlated with the levels of FT3 and FT4.

## 1. Introduction

The first case of novel coronavirus infection was reported in January 2020 in Tianjin. This sudden outbreak of infectious coronavirus disease 2019 (COVID-19) affected our lives and work. Various measures [1] were applied to prevent and control the disease progress to minimize the impact of the pandemic in China. The results of this fight against the pandemic were very obvious, and the number of cases dropped rapidly in a short period. Stress is a kind of emotional state, nonspecific systemic reactions caused by a dangerous and unexpected external condition that could cause endocrine abnormalities. Stress is a strong contributor to metabolic syndrome. Early-life stress due to deficient maternal care alters the hypothalamus-pituitary-adrenal (HPA) axis reactivity to stress [2]. Patients with thyrotoxia are generally characterized by mood disorders, and the incidence of Graves' disease in patients with anxiety disorder is higher than that of the control group, with sex differences [3]. This study aimed to explore the changes in thyroid function under the stress of this sudden pandemic.

## 2. Materials and Methods

### 2.1. Sample Collection

**Figure 1:**
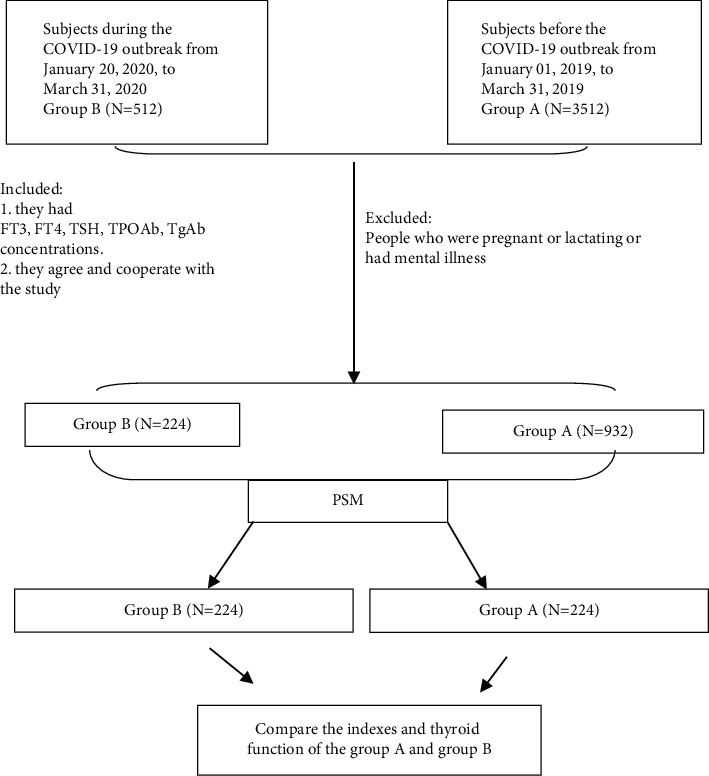
Flowchart illustrating the subjects' selection and data availability.

A total of 1395 eligible people, 932 in Group A, 224 in Group B, and 239 in Group C (Figure 1), all of whom had free thyroxine (FT4), free triiodothyronine (FT3), thyrotropin (TSH), thyroid peroxidase antibody (TPOAb), and thyroglobulin antibody (TgAb) concentrations and had no COVID-19 symptoms (such as fever, cough, and nucleic acid tests and imaging tests for COVID-19 being negative), were considered for inclusion. The exclusion criteria were pregnant or lactating women or people having mental illness. Ethical approval was obtained from the Tianjin 4th Center Hospital institutional review board (SZXLL-2020-KY0301).

All data, including sociodemographic and clinical data, were collected by doctors and nurses and recorded in the electronic medical file. Thyroid hormones, including FT3, FT4, TSH, TPOAb, and TgAb, were measured by taking fasting blood samples from the cubital vein and centrifugation within 6 hours to separate the serum, which was then tested with a LIAISON XL Automatic chemiluminescence analyzer (Saluggia, Italy). Thyroid function measurements above the normal range were considered positive. Basic diseases included cardiovascular and cerebrovascular diseases (cerebral infarction, cerebral ischemia, coronary heart disease, etc.), respiratory disease, hematologic diseases, tumors, and other medical history. An existing history of thyroid disease was defined as thyrotoxia, hypothyroidism, thyroid nodules, and thyroid autoimmune diseases. The Hamilton Anxiety Rating Scale (HAMA), consisting of 14 items, was widely used to assess the severity of anxiety.

### 2.2. Statistical Analysis

Variables are described as mean and standard deviation, and *t*-tests were used for normally distributed variables. For nonnormally distributed variables, we used the median and interquartile range; categorical variables were expressed as proportions and percentage. We used the Mann–Whitney *U* test to compare the median differences of continuous variables and the chi-square test to compare the differences of proportions. We performed a correlation analysis of the association between thyroid dysfunction and all other factors. A two-sided *p* value of less than 0.05 was considered statistically significant. All statistical tests were conducted using IBM SPSS version 24.0.

## 3. Results

### 3.1. Baseline Data

Those who met the inclusion criteria were enrolled, and among the 1395 eligible people, 932 were in Group A and 224 were in Group B. Compared to Group A, there were no significant differences in age (58.97 ± 16.91 vs. 57.51 ± 15.89, *t* = 1.222, *p*=0.222) or underlying diseases (47.8% vs. 46.4%, *χ*^2^ = 0.145, *p*=0.703). However, there were fewer female (62.1% vs. 72.3%, *χ*^2^ = 9.117, *p* < 0.01), fewer history of thyroid disease (36.6% vs. 44.7%, *χ*^2^ = 4.872, *p* < 0.05), and more abnormal diagnoses of thyroid function (69.6% vs 56.7%, *χ*^2^ = 12.615, *p* < 0.01) in Group B. With propensity score matching, 224 people exposed to the COVID-19 outbreak (Group B) were matched with 224 people in Group A. After matching, the *p* values of all variables were greater than 0.05, indicating that there were no significant differences between Group A and Group B in age, sex, and medical history of thyroid disease and basic disease. However, there was a decline in the rate of abnormal diagnoses of thyroid function in group B (69.64% vs 93.30%, *χ*^2^ = 41.539, *p* < 0.01) (Table 1).

### 3.2. Analysis of Thyroid Function

Compared with Group A, after the PSM, normal subjects (30.36% vs. 6.70%, *χ*^2^ = 41.539, *p* < 0.01) were more and hypothyroidism (14.29% vs. 35.71%, *χ*^2^ = 27.429, *p* < 0.01) and antibodies (25.89% vs. 38.39%, *χ*^2^ = 8.023, *p* < 0.01) were decreased in the pandemic Group B. There were no significant differences in thyrotoxemia (12.05% vs. 6.70%, *χ*^2^ = 3.783, *p*=0.052) and nonthyroid illness syndrome (17.41% vs. 12.50%, *χ*^2^ = 2.124, *p*=0.145). In the NTIS, the proportion of basic disease was higher in Group B than in Group A (92.30% vs. 67.90%, *χ*^2^ = 6.627, *p*=0.01). Compared with Group A, the level of FT4 (241.75 vs. 207.25, *z* = −2.821, *p* < 0.01), HAMA (7.63 ± 2.07 vs. 5.40 ± 1.65, *t* = 16.873, *p* < 0.01) was higher in Group B; however, FT3 (205.81 vs. 243.19, *z* = −3.089, *p* < 0.01), TSH (192.46 vs. 256.54, *z* = −5.238, *p* < 0.01), TgAb (152.25 vs. 296.75, *z* = −11.814, *p* < 0.01), and TPOAb (167.65 vs. 281.35, *z* = −9.299, *p* < 0.01) were lower (Table 2). HAMA score was positively related to FT3 (*r* = 0.208, *p* < 0.01) and FT4 (*r* = 0.247, *p* < 0.01), and there was no significant correlation with TSH (*r* = 0.013, *p*=0.777).

Considering the influence of thyroid disease on the diagnostic results, we did an analysis in the population without thyroid history, and compared with Group A, normal subjects (30.29% vs. 10.56%, *χ*^2^ = 16.986, *p* < 0.01) were increased and the proportion of nonthyroid illness syndrome was increased (26.76% vs. 16.9%, *χ*^2^ = 4.044, *p* < 0.05). The rate of hypothyroidism (11.97% vs. 33.8%, *χ*^2^ = 19.173, *p* < 0.01) and antibody (19.72 vs. 31.69%, *χ*^2^ = 5.329, *p*=0.021) were also decreased in the pandemic Group B. There were no significant differences in thyrotoxemia (11.27% vs. 7.04%, *χ*^2^ = 1.524, *p*=0.217) compared with Group A, and the level of FT4 (154.29 vs. 130.71, *z* = –2.418, *p* < 0.05) and HAMA score (7.51 ± 2.22 vs. 5.54 ± 2.03, *t* = 7.824, *p* < 0.01) were higher in Group B, while TSH (124.19 vs. 160.81, *z* = −3.758, *p* < 0.01), TgAb (93.97 vs. 187.07, *z* = −9.146, *p* < 0.01), and TPOAb (108.24 vs. 176.765, *z* = −7.034, *p* < 0.01) were also lower and there was no difference in the level of FT3 (135.29 vs. 149.71, *z* = −1.488, *p*=0.137) (Table 3). HAMA was positively related to FT3 (*r* = 0.245, *p* < 0.011) and FT4 (*r* = 0.292, *p* < 0.01), but there was no obvious correlation with TSH (*r* = 0.038, *p*=0.528) (Table 4).

## 4. Discussion

An increase in the number of people with abnormal thyroid function among those infected with COVID-19 has been reported around the world. Thyroid function during COVID-19 has been emphasized, and the research in this area is increasing gradually. Some studies have reported subacute thyroiditis (SAT) associated with COVID-19 [4, 5]. A retrospective study [6] by Lania et al. found evaluated thyroid function at hospitalization. Among all patients, 20.2% had thyrotoxicosis and 5.2% had hypothyroidism, and they found that thyrotoxicosis was associated with rising interleukin-6 levels, which indicates that COVID-19 may be associated with a high risk of thyrotoxicosis related to systemic immune activation including IL-1, natural killer (NK) cells [7, 8], IL-6, T-cell-mediated immune responses, and proinflammatory cytokines [9–11], while the postviral inflammatory reaction may be an inducing factor of thyroid dysfunction. At the same time, the number of studies on COVID-19-related stress is increasing. Among the 3,134 subjects studied by Sun et al., 15.5% (487 cases) showed COVID-19-related stress response, 24.9% (779 cases) showed anxiety symptoms, 28.7% (899 cases) showed depression symptoms, and 30.9% (968 cases) showed insomnia symptoms, and their proportion of detection increased compared with that before the pandemic (*p* < 0.01). The stress response is closely related to mood and insomnia aggravation [12].

Trying to manage the dramatic spread of COVID-19, Wuhan imposed a citywide lockdown and so did Tianjin since the first case of COVID-19 was diagnosed, radically changing the routine life of humans. We hypothesized that both the pandemic per se and the consequent sociopsychological sequelae could constitute stressors for the Tianjin population, potentially affecting the endocrine system. This study was designed to describe the effect of stress on the thyroid in the population north of Tianjin. A retrospective clinical trial was carried out, including inpatients and outpatients, before and during the COVID-19 pandemic. We found that people exposed to the outbreak had lower risk in proportion of thyroid abnormalities but were higher in FT4 and lower in TSH and had a higher score on the HAMA. We analyzed that the elevation of FT4 was positively associated with stress, and the decrease of FT3 and TSH was associated with chronic history and stress. This could be found in other studies as well. A prospective, observational clinical trial on the impact of COVID-19-related national lockdown on thyroid hormone in young men was conducted, and in the post-lockdown period, a significant TSH reduction was detected in 26 patients (83.9%) (*p*=0.015), whereas other hormones were not observed in terms of significant changes [13]. Abnormalities of thyroid function have also been found in special populations, and pregnant women were having higher FT3 and lower FT4 concentrations during COVID-19 [14]. Wang et al. [15] found that the levels of total triiodothyronine (TT3) and TSH were lower in COVID-19 patients than in a healthy group. Postmortem examinations have been performed in some clinical studies in patients who died of SARS-CoV-2 infection, reporting changes in the thyroid pathology, mentioning lymphocytic infiltration in the interstitium [6]. In short, thyroid function was affected not only in COVID-19 patients but also in non-COVID-19 patients during the disease outbreak and citywide lockdown, which was related to stress. Thyroid hormone was increasing in risk factors [16]. The rise of FT4 in Takotsubo syndrome suggested a stress-dependent endocrine response [17]. A meta-analytic also showed that TT3 and FT3 in patients with posttraumatic stress disorder (PTSD) were elevated compared to the control group suggesting that PTSD affected the function of the thyroid gland [18]. There are many scholars looking for causes in this area, and some related mechanisms have been found. External environmental stress causes changes in the body's internal environment, and some factors are involved, including dysfunction of the HPT axis, cellular oxidative stress, and immune molecular regulation disorders related to thyroid [19, 20]. The neuroendocrine stress axis played a pivotal role by upregulating production of the stress hormone corticosterone (CORT) to promote the release of thyroid and cortisol-related hormones [21]. The response after treatment also confirmed the correlation between stress and thyroid dysfunction again. Cellular stress [22] was abolished by treatment of antithyroid drug. Resveratrol (RSV) alleviates tunicamycin (TM)-induced ER stress by attenuating expression of genes involved in thyroid hormone synthesis in FRTL-5 thyrocytes [23]. Yuan Lili found that the HAMA scores in the thyrotoxic group were significantly higher than those in the subclinical thyrotoxic group and subclinical hypothyroidism group (*p*=0.042; *p*=0.025); HAMA scores were significantly positively related to T3 and T4 (*r* = 0.158, *p*=0.040; *r* = 0.169, *p*=0.028) [24]. A study found that employed women had a significantly higher stress burden than lactating nonemployed women, which best explained the differences in T3 suppressed [25]. However, other studies have reported that TSH increases with stress without any increase in thyroid hormones. Fischer et al. [26] found that TSH concentration was ascending in the Trier Social Stress Test (TSST), with a peak observed 20 min after the first stimulus and then after that a steady decline. No such response was observed in the control group. Posttraumatic stress disorder (PTSD) is associated with a higher risk of hypothyroidism in a dose-dependent manner in women [27]. The differences in these results may be due to differences in the detection methods of stress, study populations, and cellular stress [28]. At the same time, other factors of thyroid disease should be considered including chronic low-level inflammation [29] and imbalance of oxidation and antioxidation [30, 31]. In an animal study, Nair Betina et al. examined hypothalamic, pituitary, and endocrine responses to 14 days of chronic variable stress (CVS) in male and female mice. The mRNA expression of pituitary TSH increased significantly during CVS as well as upregulation of the neuroendocrine stress axis [32]. At the gene level, the study of thyroid hormones and immune cells has gradually increased. TREM2, its expression in macrophages and microglia, is stimulated by thyroid hormone [33]. Thyroid hormone agonism was also found to induce phagocytic behavior in microglia consistent with the TREM2 pathway.

The proportion of NTIS in the pandemic period increased compared with the control group, especially in the population without a thyroid disease history. This is related to the condition of the patients who visited the hospital during COVID-19. Lui [34] found that patients with COVID-19 were more likely to have isolated low FT3, with normal TSH and FT4 levels, suggesting a possible NTIS. NTIS is usually associated with disease severity and deterioration prognosis in critical illness. A research by Wei et al. analyzed thyroid function and pathological changes in patients with SARS-CoV-2. Even after a few years, the serum TSH level of COVID-19 patients was lower than that of healthy controls; regular assessment of thyroid status may be helpful [35, 36]. We will continue to follow the changes and research in the future.

## 5. Conclusion

People in the northern area of Tianjin during the COVID-19 outbreak were at an increased risk of higher FT4, lower FT3, and lower TSH. HAMA scores increased in emergency situations and were positively correlated with the levels of FT3 and FT4. Further prospective study was needed to know the cause and effect relation between them.

## Figures and Tables

**Table 1:** Changes in thyroid function during the pandemic.

Assessment characteristics		Before matching				After matching			
		Group A (*N* = 932)	Group B (*N* = 224)	Group B vs A		Group A (*N* = 224)	Group B (*N* = 224)	Group B vs A	
				*t* or *χ*^2^ value	*p*			*t* or *χ*^2^ value	*p*
Gender	Male	258 (27.68%)	85 (37.95%)	9.117^A^	<0.01	85 (37.95%)	85 (37.95%)	0^A^	1
	Female	674 (72.32%)	139 (62.05%)			139 (62.05%)	139 (62.05%)		

Age (year)		57.51 ± 15.89	58.97 ± 16.91	1.222^B^	0.222	58.61 ± 16.18	58.97 ± 6.91	0.234^B^	0.815

Basic disease^1^	Yes	432 (46.35%)	107 (47.76%)	0.145^A^	0.703	98 (43.75%)	107 (47.76%)	0.728^A^	0.393
	No	500 (53.65%)	117 (52.24%)			126 (56.25%)	117 (52.24%)		

History of thyroid	Yes	417 (44.74%)	82 (36.61%)	4.872^A^	<0.05	82 (36.61%)	82 (36.61%)	0^A^	1
	No	515 (55.26%)	142 (63.39%)			142 (63.39%)	142 (63.39%)		

Diagnosis of thyroid function	Normal	404 (43.35%)	68 (30.36%)	12.615^A^	<0.01	15 (6.70%)	68 (30.36%)	41.539^A^	<0.01
	Abnormal	528 (56.65%)	156 (69.64%)			209 (93.30%)	156 (69.64%)		

Note: ^1^basic diseases include coronary heart disease, cerebral infarction, cerebral ischemia, respiratory disease, hematologic disease, tumor, and other medical history, Group A: control group; Group B: COVID-19 outbreak group; A: chi-square test; B: *t*-test.

**Table 2:** Comparison of thyroid function between Group A and Group B.

Thyroid function	Group A, *N* (%)	Group B, *N* (%)	Value	*p*
Hypothyroidism	80 (35.71%)	32 (14.29%)	27.429^A^	<0.01
Without hypothyroidism	144 (64.29%)	192 (85.71%)		
Thyrotoxia	15 (6.70%)	27 (12.05%)	3.783^A^	0.052
Without thyrotoxia	209 (93.30%)	197 (87.95%)		
Normal	15 (6.70%)	68 (30.36%)	41.539^A^	<0.01
Abnormal	209 (93.30%)	156 (69.64%)		
TgAb or TPoAb positive	86 (38.39%)	58 (25.89%)	8.023^A^	<0.01
TgAb or TPoAb negative	138 (61.61%)	166 (74.11%)		
NTIS	28 (12.50%)	39 (17.41%)	2.124^A^	0.145
Without NTIS	196 (87.50%)	185 (52.59%)		
HAMA	5.40 ± 1.65	7.63 ± 2.07	16.873^B^	<0.01
FT3	5.00 (4.00, 6.00)	4.95 (3.64, 5.60)	−3.089^C^	<0.01
FT4	13.74 (11.48, 15.97)	14.52 (12.35, 16.86)	−2.821^C^	<0.01
TSH	3.58 (1.62, 7.74)	2.30 (1.03, 3.95)	−5.238^C^	<0.01
TgAb	481.40 (111.9, 1440.5)	15.97 (5.38, 101.93)	−11.814^C^	<0.01
TPOAb	265.5 (34.52, 1087.75)	15.97 (5.38, 101.93)	−9.299^C^	<0.01

A: chi-square test; B: t-test; C: Mann–Whitney U test.

**Table 3:** Comparison of thyroid function between Group A and Group B without thyroid disease.

Thyroid function	Group A, *N* (%)	Group B, *N* (%)	Value	*p*
Hypothyroidism	48 (33.80%)	17 (11.97%)	19.173^A^	<0.01
Without hypothyroidism	94 (66.20%)	125 (88.03%)	Value	*p*
Thyrotoxia	10 (7.04%)	16 (11.27%)	1.524^A^	0.217
Without thyrotoxia	132 (92.96%)	126 (88.73%)		
Normal	15 (10.56%)	43 (30.29%)	16.986^A^	<0.01
Abnormal	127 (98.44%)	99 (69.71%)		
TgAb or TPoAb positive	45 (31.69%)	28 (19.72%)	5.329^A^	<0.05
TgAb or TPoAb negative	97 (68.31%)	114 (80.28%)		
NTIS	24 (16.90%)	38 (26.76%)	4.044^A^	<0.05
Without NTIS	118 (83.10%)	104 (73.24%)		
HAMA	5.54 ± 2.03	7.51 ± 2.22	7.824^B^	<0.01
FT3	5.00 (3.28, 6.00)	4.36 (3.33, 5.00)	−1.488^C^	0.137
FT4	13.80 (11.16, 16.20)	14.63 (12.39, 16.85)	−2.418^C^	0.016
TSH	3.581 (1.49, 8.38)	2.30 (1.12, 3.97)	−3.758^C^	<0.01
TgAb	228.45 (48.41, 752.45)	12.47 (7.87, 55.05)	−9.146^C^	<0.01
TPOAb	198.1 (22.49, 946.45)	13.6 (4.84, 83.97)	−7.034^C^	<0.01

A: chi-square test; B: *t*-test; C: Mann–Whitney *U* test.

**Table 4:** Correlation analysis between HAMA and thyroid hormone in patients without thyroid disease by Pearson analysis.

Thyroid hormone	OR	*p*
FT3	0.245	<0.01
FT4	0.292	<0.01
TSH	0.038	0.528

## Data Availability

The SAV data used to support the findings of this study are included within the article and the supplementary information files. These data are currently under embargo while the research findings are commercialized. Requests for data, 6 months after publication of this article, will be considered by the corresponding author.
