# Corrigendum to “Thyroid Hormone Changes in the Northern Area of Tianjin during the COVID-19 Pandemic”

**DOI:** 10.1155/2022/9757320

**Published:** 2022-08-16

**Authors:** Dong Weiwei, Wu Bei, Wang Hong, Wu Cailan, Shao Hailin, Xu Donghong, Wang Xiaolai, Hao Zhaohu, Li Shijun, Tan Jian, Jia Qiang

**Affiliations:** ^1^Department of Nuclear Medicine, Tianjin Fourth Central Hospital, The Fourth Central Clinical School, Tianjin Medical University, Tianjin 300140, China; ^2^Department of Nuclear Medicine, Tianjin Medical University General Hospital, Tianjin Medical University, Tianjin 300052, China; ^3^Department of Nuclear Medicine, Tianjin Hospital, Tianjin 300211, China; ^4^Rehabilitation Medical Department, Tianjin Union Medical Center, Tianjin 300121, China; ^5^Department of Endocrinology Medicine, Tianjin Fourth Central Hospital, The Fourth Central Clinical School, Tianjin Medical University, Tianjin 300140, China; ^6^Department of Hematology, Tianjin Fourth Central Hospital, The Fourth Central Clinical School, Tianjin Medical University, Tianjin 300140, China

In the article titled “Thyroid Hormone Changes in the Northern Area of Tianjin during the COVID-19 Pandemic” [1], the affiliation of Tan Jian was previously listed incorrectly as ^1^Department of Nuclear Medicine, Tianjin Fourth Central Hospital, The Fourth Central Clinical School, Tianjin Medical University, Tianjin 300140, China. The correct affiliation is ^2^Department of Nuclear Medicine, Tianjin Medical University General Hospital, Tianjin Medical University, Tianjin 300052, China. The affiliation has been corrected as above.
